# The Faucial Tonsil as a Focus for Systemic Infection

**Published:** 1913-09

**Authors:** George E. Shambaugh

**Affiliations:** Chicago


### The Faucial Tonsil as a Focus for Systemic Infection.

Formerly it was assumed that sore throat occurring in connection with acute rheumatism was but a local manifestation in the pharynx of the general systemic infection. It is now generally believed that sore throat in these cases represents the focus of entrance for the systemic infection. Furthermore, it is generally recognized that sore throat, for the most part acute tonsilitis, is very often the immediate cause for other systemic infections, such as acute endocarditis and acute nephritis. It has not been so generally appreciated that the faucial tonsils are very frequently the foci for chronic systemic infections, such as chronic arthritis, chronic neuritis, cardiovascular degeneration, and chronic nephritis. The general practitioner as well as the specialist has not fully appreciated the importance of the relation existing between infections, acute as well as chronic, of the faucial tonsils and certain systemic conditions. Very frequently in ill nourished children the removal of the tonsils results in such an immediate and astonishing improvement in general health that it can hardly be accounted for except on the assumption that the enlarged harmless looking tonsils contained foci for a mild systemic infection. In many of the tonsils there are dilated crypts containing the characteristic cheesy deposits which from time to time produce acute infection. The small tonsil embedded under a fold from the anterior pillar, and the tonsil with a deep horizontal fissure separating the upper from the middle thirds, are unusually susceptible to acute infections and are especially predisposed to the development of latent foci capable of causing systemic infections. Another type of faucial tonsil which is a frequent source of systemic infection is the stub remaining after partial removal, or where the tonsil has been subjected to igni puncture or surface cauterization. The treatment of a faucial tonsil suspected of harboring foci of infection is the same as such foci elsewhere in the body, namely, thorough removal of the suspected foci.—Dr. George E. Shambaugh, Chicago.

